# Expression of *osterix* Is Regulated by FGF and Wnt/β-Catenin Signalling during Osteoblast Differentiation

**DOI:** 10.1371/journal.pone.0144982

**Published:** 2015-12-21

**Authors:** Katharina Felber, Philip M. Elks, Maria Lecca, Henry H. Roehl

**Affiliations:** Bateson Centre and Department of Biomedical Science, University of Sheffield, Sheffield S10 2TN, United Kingdom; Kyungpook National University School of Medicine, REPUBLIC OF KOREA

## Abstract

Osteoblast differentiation from mesenchymal cells is regulated by multiple signalling pathways. Here we have analysed the roles of Fibroblast Growth Factor (FGF) and canonical Wingless-type MMTV integration site (Wnt/β-Catenin) signalling pathways on zebrafish osteogenesis. We have used transgenic and chemical interference approaches to manipulate these pathways and have found that both pathways are required for osteoblast differentiation *in vivo*. Our analysis of bone markers suggests that these pathways act at the same stage of differentiation to initiate expression of the osteoblast master regulatory gene *osterix* (*osx*). We use two independent approaches that suggest that *osx* is a direct target of these pathways. Firstly, we manipulate signalling and show that *osx* gene expression responds with similar kinetics to that of known transcriptional targets of the FGF and Wnt pathways. Secondly, we have performed ChIP with transcription factors for both pathways and our data suggest that a genomic region in the first intron of *osx* mediates transcriptional activation. Based upon these data, we propose that FGF and Wnt/β-Catenin pathways act in part by directing transcription of *osx* to promote osteoblast differentiation at sites of bone formation.

## Introduction

The bony skeleton initially develops in one of two ways, either by ossification of a cartilage template (chondral ossification) or in the absence of a cartilage template (achondal ossification). Osteoblasts (specialised cells that synthesise bone) are derived from multipotent mesenchymal stem cells which are found in different tissues. During chondral bone development, osteoblasts initially differentiate in the perichondrium (a tissue which surrounds the cartilage), and in achondral bone development osteoblasts differentiate in mesenchymal cell condensations. Later on in development and in adults, osteoblast progenitors are found in the bone marrow as well as in the periosteum (a tissue which surrounds bone). Genetic analysis in mice has identified two transcription factors, *Runx2* (*Runt-related transcription factor 2*) and *Osx* (also known as *Sp7*), that act in a transcriptional cascade during osteoblast differentiation. In mice lacking either gene osteoblasts throughout the body fail to differentiate while other cell types are largely unaffected[1–3]. In osteoblast progenitors, *Runx2* expression precedes that of *Osx* and it is known that Runx2 is required to activate *Osx* transcription[4]. Both transcription factors have been shown to activate expression of many markers of mature osteoblasts including *Collagen 1*(*Col1*), *Secreted protein acidic cysteine-rich* (*SPARC*), *Osteopontin* (*Opn*), *Bone Sialoprotein* (*BSP*), *Osteocalcin* (*Osc*) and *Alkaline phosphatase* (*ALP*)[5,6]. Whether *Osx* also plays a pivotal role in osteoblastogenesis in humans is unclear as one study suggests a relatively mild skeletal phenotype occurs when *OSX* is mutated[7].

FGF signalling plays a crucial role during skeletal development. Mutations in human *FGFR1*, *FGFR2*, *FGFR3*, *FGF10* and *FGF23* all cause skeletal defects consistent with a role in osteoblast differentiation and/or function[8]. However, experiments to define the role of the FGF pathway in osteoblastogenesis have often generated conflicting results (reviewed in [9] and [5]). For example it has been shown that FGF signalling activates expression of *Runx2* in MSCs to initiate the osteoblast lineage, and later activates Opn and BSP expression in maturing osteoblasts [10–14]. This is supported by in vivo studies that have shown that mutations that impair FGF sigalling reduced bone density [15–17]. On the other hand, activation of FGF signalling in vitro results in reduced expression of *ALP* and *Col1* and induces osteoblast apoptosis [18–20]. These results suggest that FGF signalling may play different roles during osteoblast differentiation and that timing and strength of the FGF signal is crucial in these outcomes.

Wnt signalling via the β-Catenin pathway has more recently been identified as a key regulator of osteoblastogenesis [21–23]. As with FGF signalling, a consensus has not emerged regarding the precise role of the Wnt/β-Catenin pathway. Conditional inactivation of *β-Catenin* in the murine embryo has established that it is required for *Osx*, *Osc* and *Col1* expression in the osteoblast lineage [24–27]. However, *β-Catenin* knock-out also causes an increase in the expression of *Runx2* and expression of a constitutively active form of β-Catenin blocks entry into the osteoblast lineage. Together these results suggest that Wnt/β-Catenin acts at two sequential stages, to inhibit differentiation initially, then to promote differentiation after commitment. Other studies using murine MSCs have found that Wnt3a treatment upregulates *ALP*, but does not affect *Runx2*, *Osc* or *Col1* levels[28,29]. Further studies have shown that Wnt/β-Catenin signalling promotes early osteoblastogenesis in vivo and in mouse embryonic fibroblasts by direct activation of *Runx2* expression [30,31]. Studies using human MSCs have found that Wnt/β-Catenin acts to suppress entry into the osteoblast lineage [32–34] and analysis of *Wnt10b* ^*-/-*^ mice suggests that an osteopenic phenotype results from decreased maintenance of adult MSC in bone [35]. The finding that *Osx* and Wnts interract in positive and negative regulatory loops may explain why it has been very difficult to ascribe a simple role for Wnts in skeletal development [36,37]. Together these studies indicate that role of Wnt/β-Catenin signalling varies according to the precise timing and context of the signalling event.

Analysis of zebrafish bone development suggests that the regulation of osteoblastogenesis is conserved between fish and mammals. As in mammals, the retinoic acid, BMP and Hedgehog pathways regulate recruitment and/or anabolic activity of osteoblasts in zebrafish [38–41]. Expression of *runx2* (*runx2a* and *runx2b*), *osx*, *col1a2*, *sparc*, *osc* and *opn* mark progression of osteoblastogenesis [40,42–46]. Reporter transgenes based upon *osx* genomic sequence have been generated in both zebrafish and medaka[46,47]. Both achondral and chondral skeletal development takes place in zebrafish, and although bone is cellular, bone marrow does not form [48–50]. Here we analyse the roles that FGF and Wnt/β-Catenin pathways play during achondral ossification in the head. We find that both pathways promote ossification and act at the level of *osx* expression. Besides acting in parallel to regulate *osx* expression, we also find evidence that Wnt/β-Catenin signalling regulates the activity of the FGF pathway during skeletogenesis.

## Materials and Methods

All methods were performed using standardised protocols[51]. All animal husbandry and experimentation was carried out under the supervision and approval of the Home Office (UK) and the University of Sheffield Ethics Board. Adult zebrafish were maintained with a 14 h light/10 h dark cycle at 28°C according to standard protocols and were mated using pair mating in individual cross tanks. To kill fish, larvae were anesthetized with 0.2 mg/ml of Tricane at 4°C. Unless otherwise stated, 10 larvae were analysed for each sample after chemical treatment or heat shock and representative images were chosen for figure panels.

### Alizarin Red staining

Larvae were fixed 2 hours (hrs) in 4% formaldehyde/PBS, stored in 80%MeOH/H2O for 1 hour. The larvae were rinsed briefly in H2O with 0.1% Tween-20 (H2Otw). Then larvae were bleached in 1.5% H2O2 in 1% KOH for 30 minutes at 37C. Larvae were stained in 1% KOH with 0.04 mg/ml Alizarin red for 2 hrs. Larvae were passed through a glycerol series (25%, 50%, 80% glycerol in H2Otw) 10 minutes each and then photographed in 80% glycerol/H2O.

### Alcian Blue staining

Larvae were fixed as above then stained in 0.1% Alcian Blue in 0.1N HCl overnight and bleached as above. The staining was fixed for 10 minutes in borate buffer (30% saturated-sodium tetraborate in H2Otw), and then cleared with trypsin (0.5mg/ml trypsin in borate buffer) until the tissue was completely digested away from the skeleton (2–3 hrs at 37°C). Larvae were then photographed in glycerol as above. Larvae stained this way can be stored indefinitely.

### von Kossa staining

Larvae fixed at 120hpf for 2 hrs in 4%PFA were rinsed 3X 5min in H2Otw. Then larvae were incubated for 45–60 minutes in 1% aqueous silver nitrate under incandescent lamp (60W) and staining was monitored at regular time intervals. Following 3X 5min H2Otw rinses larvae were then fixed in 2.5% sodium thiosulfate for 10 minutes and post-fixed in 4%PFA for 30 minutes. Larvae were then photographed in glycerol as above. Larvae stained this way can be stored indefinitely.

### Heat shock procedure

20–40 larvae were placed in 25ml E3 buffer in a 50ml falcon tube and put into a water bath at 38°C for one hr. Heat shock time refers to the start of the heat shock. Continuous transgenic expression was accomplished by heat shocking for one hour every 12 hours for the duration of the treatment. All experiments were done with hemizygous carriers and included the wild type siblings as controls, larvae were sorted after heat shock based upon fluorescence of the transgene.

### Chemical treatments

SU5402 (Merck) was made up in DMSO to 10mM and stored at -20°C. GSK-3 Inhibitor XV (Merck) was made up in DMSO to 5mM and stored at -20°C. All treatments were done at a dilution of 10uM in E3 in the dark.

### Time courses and qPCR

For FGF signalling, three batches of 15 wild type embryos were treated with SU5402 at 50hpf(2hrs), 50.5hpf(1.5hrs), 51hpf(1hr) and 51.5hpf(0.5hr). All treatments and an untreated batch were terminated at 52hpf and processed using Trizol and Superscript II following the manufacturer's protocols (Invitrogen). qPCR was performed using iQ SYBR Green Supermix (Bio-Rad) with a Bio-Rad MyiQ system. Primer pairs for *pea3* and *osx* performed equivalently in a dilution series so the comparative C_T_ method was used to calculate relative expression levels. For Wnt/β-Catenin signalling, *hs*:*dkk1* hemizygous fish were crossed to *Tg(TOP*:*GFP)* homozygous carriers. The offspring were heat shocked at 48hpf and sorted based upon fluorescence. Three batches of fifteen *hs*:*dkk1*and fifteen sibling fish were fixed every two hours until 54hpf. RNA was processed using Ultraspec (AMS Biotechnology) and the High Capacity cDNA Reverse Transcription Kit (Applied Biosystems) using manufacturers' protocols. Input levels were quantified using TaqMan probes, and Universal Mastermix and ABI 7200 (Applied Biosystems). The housekeeping gene *phosphoglycerate kinase 1* (*pgk1*) was used to normalise. Non-heatshocked fish at 48hpf (unsorted) were also processed as a control. Significance was tested using the Students T-test using three technical replicates and comparison of the individual dCT values. Primer sequences are listed in S1 Table.

### Chromatin Immunoprecipitation (ChIP)

100 embryos were first deyolked using 1ml deyolking buffer and vortexed at 1000rpm for 5 minutes, cross-linked in 1ml fresh 1% formaldehyde for 15 minutes at room temperature and washed 3 times with 0.125M Glycine and 2 times with cold PBS. DNA was extracted by adding 600μl DNA extraction buffer (containing protease inhibitors), mixed 20 times with a 200μl tip and incubated on a shaker for 1 to 2 hours at 4°C. After centrifugation for 5 minutes at 3500rpm (4°C), the pellet was dissolved in 400μl IP dilution buffer and the DNA was sonicated. A sample of the DNA was analysed by gel electrophoresis to ensure a fragment size of approximately 200–1000 base pairs. 40μl of washed Protein A bead slurry was added and rotated at 4°C for 1 hour. The beads were removed and the antibodies (and input containing no antibody) were added and the sample was rotated over night at 4°C: β-catenin (Sigma C2206) 10μl; ETS 1/2 (Santa Cruz C-275) 25μl. 40μl of beads were added to each sample and the mix was incubated for 1 hour at room temperature. The beads were washed and elution buffer was added (150μl) to the beads and rotated for 15 minutes at room temperature. The elution step was repeated once more. Thereafter the beads were discarded and 22.5μl 4M NaCl and 1μl RNase A was added to the eluate and this was incubated 5 hours at 65°C. The sample was then precipitated, the pellet was dissolved in and incubated with proteinase K at 45°C for 3 hours. After Phenol/Chloroform extraction, the sample was precipitated again, and cleaned with the PCR purification kit (Qiagen). It was eluted in 15μl TE buffer and stored at -20°C. For a detailed protocol please contact the authors. Primer sequences are listed in S1 Table.

### Quantification of in situ stainings

To quantify the staining, three independent sets (>10 fish each) were treated with DMSO, SU5402, hsdnFGFR1 or hsFGF3 and processed identically for each experiment. These were then scored for the presence of staining associated in the opercle, and the data was combined to generate an average with associated standard deviation. As loss of staining was generally all or nothing, animals were scored for the presence of any staining (scored as one) or the complete loss of staining (scored as 0). The researcher was blind to which sample was control or treated during this analysis.

## Results

### FGF signalling is required for ossification and the development of mature osteoblasts

**Fig 1:**
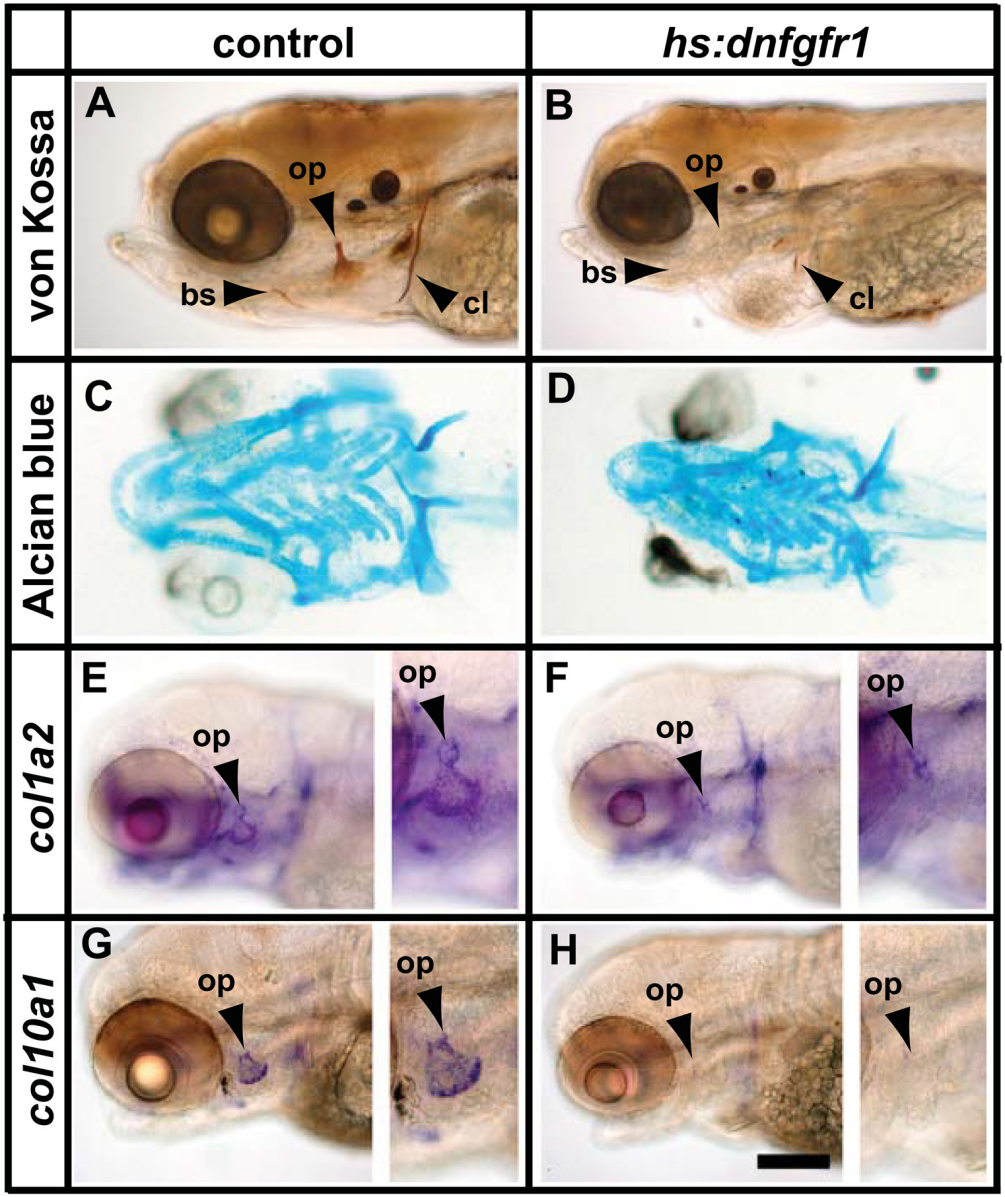
FGF signalling is required for ossification and osteoblast maturation. (A-D) Continuous inhibition of FGF signalling from 48hpf to 120hpf in *hs*:*dnfgfr1* larvae results in loss of ossification (B) but cartilage formation is relatively unaffected (D). (E-H) Expression of mature osteoblast markers *col1a2* and *col10a1* is severely reduced in *hs*:*dnfgfr1* larvae (F,H). High magnification images of the opercle are shown to the right of panels E-H. Abbreviations: bs = branchiostegal ray, cl = cleithrum, op = opercle. Scale bar = 200μM.

To determine whether FGF signalling acts during zebrafish bone development we took advantage of a zebrafish transgenic line called *hs*:*dnfgfr1* that expresses a dominant negative form of the FGF under control of a heat shock promoter (*Tg(hsp70l*:*dnfgfr1-EGFP*); [52]). Hemizygous fish were continuously exposed to dnFGFR1 from 48 hours post fertilisation (hpf) until 120hpf (heat shock for one hr every eleven hours). This treatment completely abolished ossification except for a small part of the cleithrum which begins to ossify before the start of the treatment (Fig 1A and 1B). Chondrogenesis, which also takes place during this time period was not noticeably affected (Fig 1C and 1D). It is possible that treatment starting at earlier timepoints would affect the cartilagenous skeleton but such treatments result in disruption of morphogenesis of the embryo (data not shown). The loss of bone could reflect defects in calcium homeostasis or in osteoblast activity. To determine whether osteoblasts were present and secreting bone matrix proteins, we performed in situ analysis with *opn*, *col10a1* and *col1a2* (Fig 1E–1H and data not shown). Although expression of *col10a1* is typically described as being associated with hypertrophic chondrocytes in mammals, it is expressed in mature osteoblasts in zebrafish[43,53]. Both markers were drastically reduced or absent in treated fish suggesting that FGF signalling is required for the differentiation or the anabolic activity of osteoblasts.

**Fig 2:**
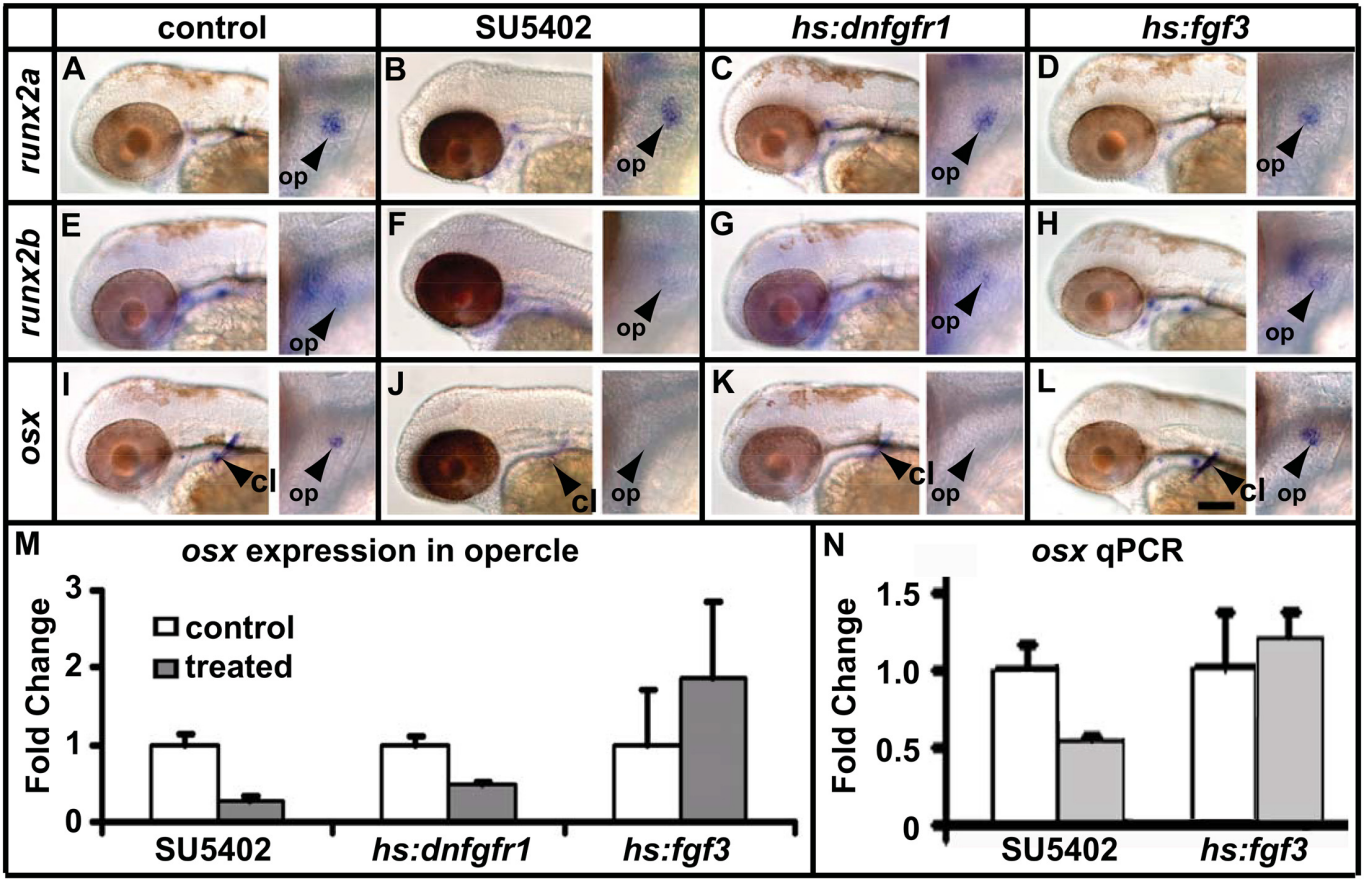
FGF signalling regulates expression of *osx*. (A-H) Larvae were treated at 48hpf, fixed at 51hpf and expression of several bone markers was analysed by in situ hybridisation. Arrowheads point to the opercle in the high magnification images to the right of each panel. Expression of *runx2a* (A-D) and *runx2b* (E-H) is unchanged after inhibition of FGF signalling using *hs*:*dnfgfr1* larvae as well as over activation in *hs*:*fgf3* transgenic fish. Expression of *runx2b* is slightly reduced after SU5402 treatment (F). (I-L) Expression of *osx* is decreased after inhibition of FGF signalling and increased after over activation of FGF signalling as shown by in situ hybridization. Abbreviations: cl = cleithrum, op = opercle. Scale bar = 200μM. (M) Quantification for presence or absence of *osx* staining in the opercle from I-L. Expression was normalised to the control. (N) qPCR performed in parallel to I-L confirm effects of FGF signalling on *osx* expression. Treatments were normalised to *gapdh*. Treatment with SU5402 showed a significant reduction (p<0.05 Student's t-test).

To test whether osteoblast differentiation requires FGF signalling we decided to analyse earlier stages, focusing primarily on development of the opercle bone. *runx2a* and *runx2b* are early markers of osteoblasts that first appear in the region of the opercle at around 48hpf followed by *osx* at around 51hpf. We used the *hs*:*dnfgfr1* line and the pharmacological inhibitor SU5402 to block FGF signalling and a *hs*:*fgf3* line to activate FGF signalling. To determine when these treatments have their strongest effect, we used expression of *pea3* (*polyomavirus enhancer activator 3*) and *erm* (*ets related molecule*) as transcriptional read-outs of the FGF pathway [54]. We found that three hours after the onset of treatment has the optimal effect on FGF signalling throughout the embryo (S1 Fig). Treatment starting at 48hpf lasting for three hours did not affect *runx2a* expression (Fig 2A–2D), and a mild reduction in *runx2b* was seen only after treatment with SU5402 (Fig 2E–2H). In contrast, *osx* expression was strongly downregulated by both *hs*:*dnfgfr1* and SU5402 treated fish and upregulated by *hs*:*fgf3* (Fig 2I–2L). To quantify these results we counted the number of fish which had staining in the opercle and did qPCR on whole larvae and both methods established that perturbation of FGF signalling has a significant effect on *osx* gene expression. To determine whether the expression of late osteoblast markers (i.e. bone matrix genes) is affected immediately following perturbation of FGF signalling, we treated fish for three hours starting at 60hpf (S2 Fig). Neither SU5402 treatment or *hs*:*fgf3* had a an affect on the expression of *col1a2* (and *sparc*, data not shown) indicating that FGF signalling is not likely to directly regulate the expression of these bone matrix genes.

### Wnt/β-Catenin signalling is required for ossification and the development of mature osteoblasts

**Fig 3:**
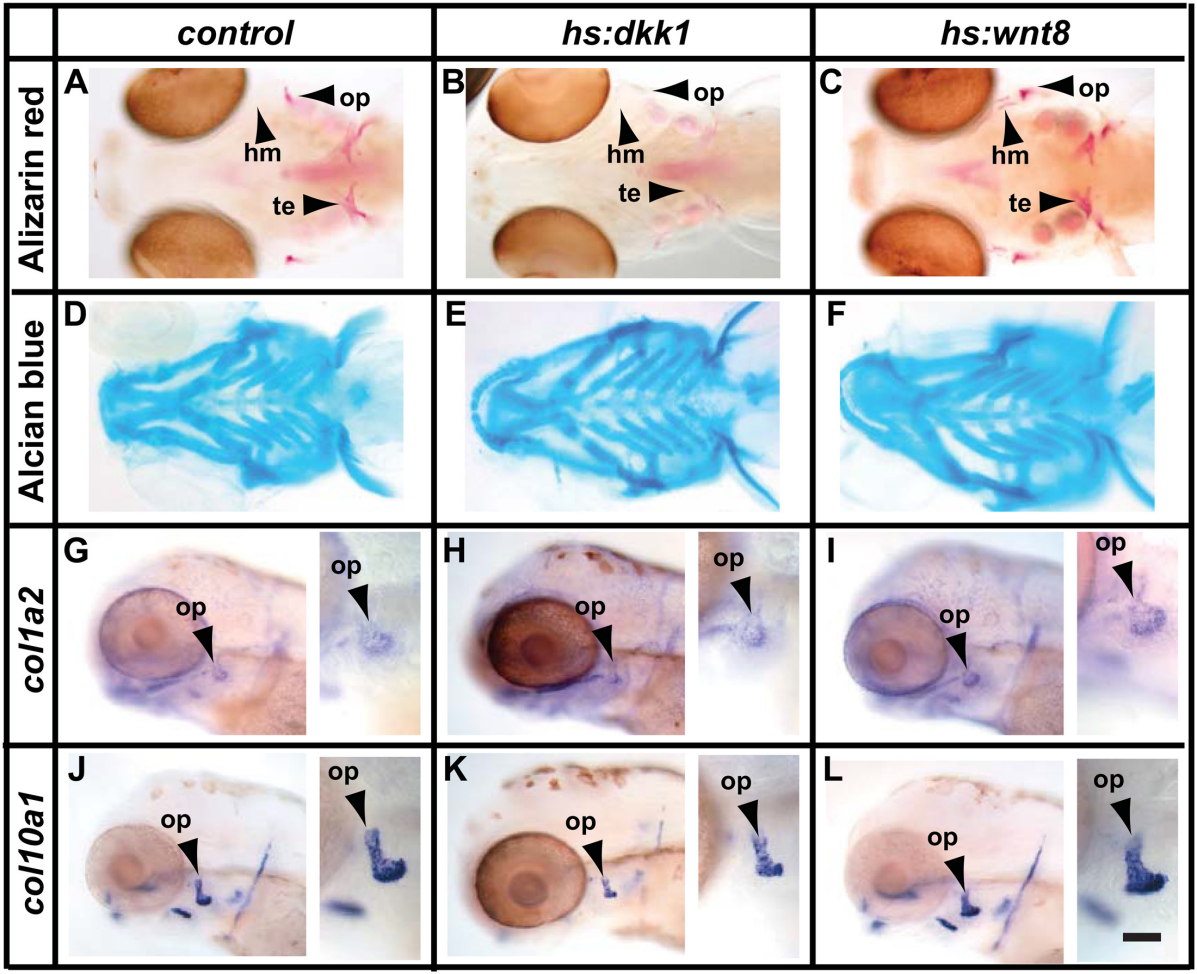
Wnt/β-Catenin signalling regulates ossification and development of mature osteoblasts. (A-F) Continuous inhibition (*hs*:*dkk1*) or over activation (*hs*:*wnt8*) of Wnt/β-Catenin signalling from 72-120hpf. Inhibition of Wnt/β-Catenin signalling results in reduced ossification as shown by Alizarin red staining (B) whereas cartilage formation is largely unaffected (E). Overactivation of Wnt/β-Catenin signalling results in increased ossification and precocious ossification of the hyomandibula (C). (G-L) Analysis of osteoblast markers after treatment from 72-108hpf. Expression of mature osteoblast markers *col1a2* and *col10a1* is slightly reduced when Wnt/β-Catenin signalling is inhibited (H,K) and enhanced by increased Wnt/β-Catenin signalling (I,L). Abbreviations: hm = hyomandibula, op = opercle, te = teeth. Scale bar = 50μM.

To determine the role of Wnt/β-Catenin signalling during osteoblast differentiation we used two transgenic lines: *hs*:*dkk1* (*Tg(hsp70l*:*dkk1-GFP*); [55]) to downregulate signalling and *hs*:*wnt8a* (*Tg(hsp70l*:*wnt8a-GFP*); [56]) to upregulate signalling. We found that continuous suppression of Wnt signalling starting at 48hpf resulted in reduced ossification levels at 120hpf (Fig 3B). Similar treatment using *hs*:*wnt8a* resulted in an increase in ossification (Fig 3C). Neither treatment resulted in a strong change to the cartilagenous skeleton (Fig 3D–3F). It is possible that treatment starting at earlier timepoints would affect the cartilagenous skeleton but such treatments resulted in disruption of morphogenesis of the embryo (data not shown). Treatment from 72hpf until 108hpf resulted in reduced *col10a1* and *col1a2* in *hs*:*dkk1*fish and increased expression in *hs*:*wnt8a* fish (Fig 3G–3L). These data are comparable to the results obtained by manipulating FGF signalling and suggest a role for Wnt/β-Catenin signalling in osteoblast differentiation or bone matrix secretion.

**Fig 4:**
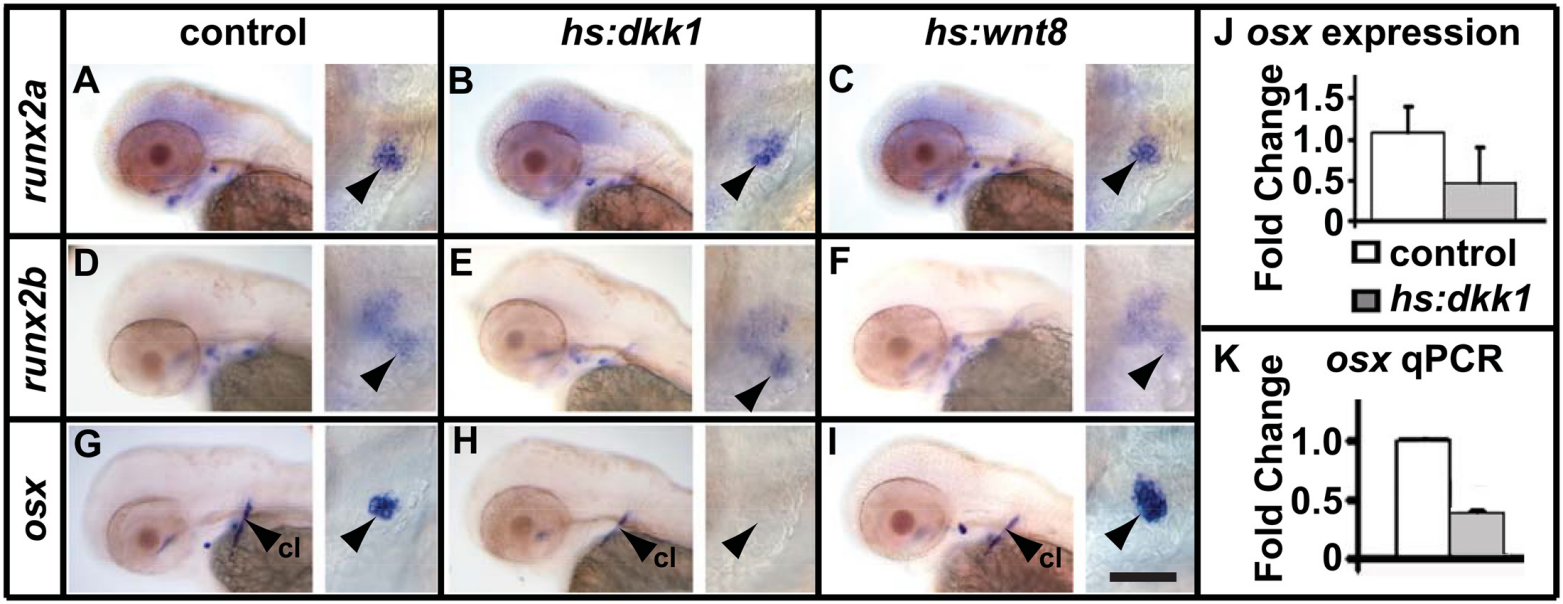
Wnt/β-Catenin signalling regulates *osx* expression. (A-I) Larvae were treated at 48hpf and fixed 12hours later. Expression of *runx2a* (A-C) and *runx2b* (D-F) is unchanged after inhibition (*hs*:*dkk1*) or over activation (*hs*:*wnt8*) of Wnt/β-Catenin signalling. (G-L) Expression of *osx* is reduced in *hs*:*dkk1* and increased in *hs*:*wnt8* larvae 12 hours after the treatment. Arrowheads point to the opercle in the high magnification images to the right of each panel. Abbreviations: cl = cleithrum. Scale bar = 50μM. (J) Quantification for presence or absence of *osx* staining in the opercle from G and H. Expression was normalised to the control. (K) qPCR performed in parallel on g and h confirms the effects of Wnt/β-Catenin signalling on *osx* expression (p<0.01 using Student's t-test). Treatments were normalised to *gapdh*.

To further investigate Wnt activity, we decided to test whether early markers of osteoblastogenesis are regulated by Wnt/β-Catenin signalling. We first assayed how rapidly the lines have the predicted effect on known Wnt/β-Catenin targets. We found that 8 hours treatment starting at 48hpf has a strong effect on expression of both *wif1* and *axin2* (S3 Fig). Next we looked at expression of *runx2a*, *runx2b* and *osx* and found that only *osx* expression was altered by heatshock treatment (Fig 4A–4I). To validate this result we used qPCR to quantify the reduction in *osx* expression after 12 hours of *dkk1* over expression and found that expression is reduced to 40% of wild type levels (Fig 4K). We also tested whether *col1a2* expression responds rapidly to Wnt/β-Catenin perturbation and found that it was unchanged (S2 Fig). Together these results suggested that Wnt/β-Catenin signalling acts to promote osteoblast differentiation and does so by activating *osx* expression.

### FGF and Wnt/β-Catenin signalling may activate *osx* expression via an intronic cis-regulatory module

**Fig 5:**
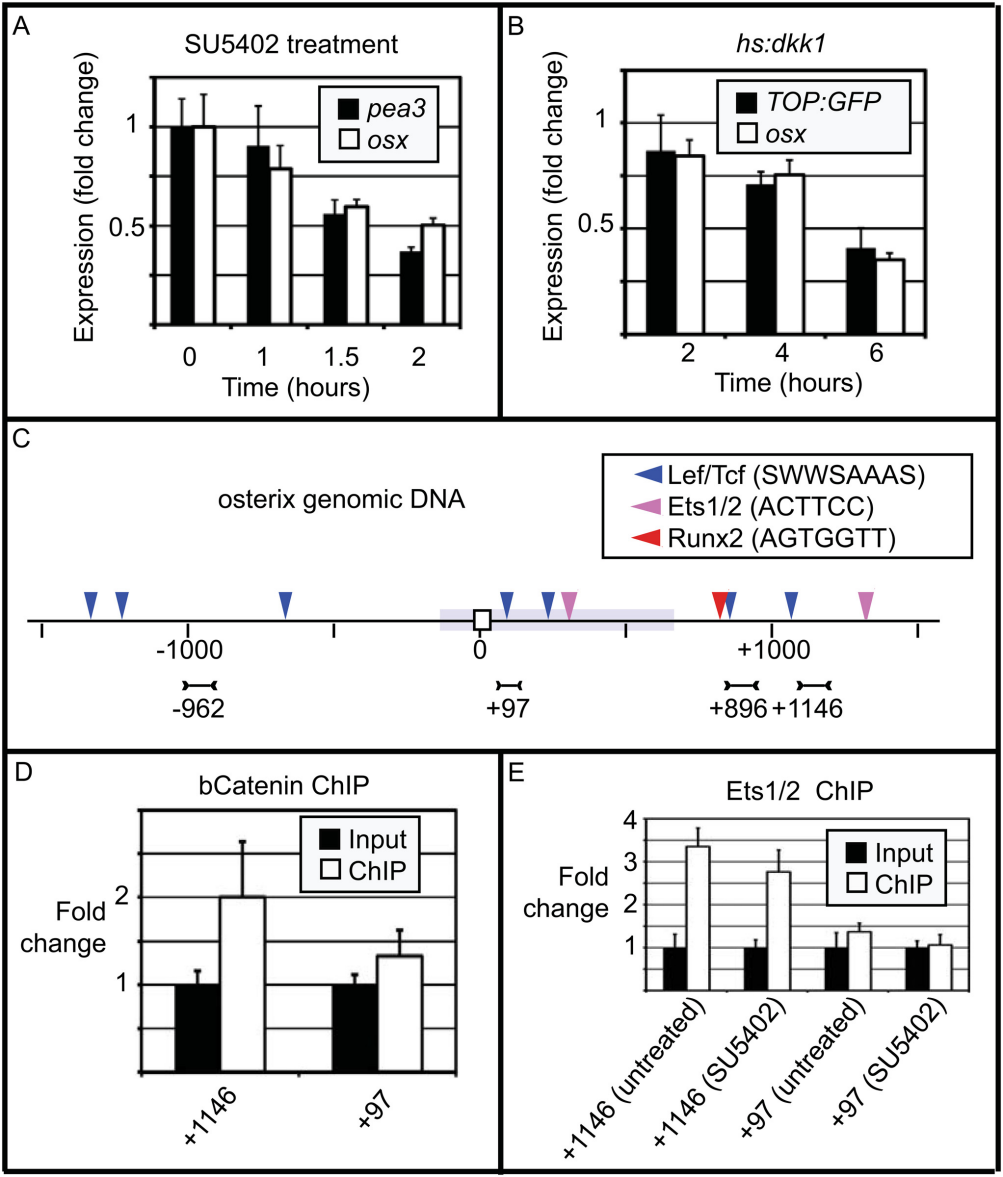
*osx* is likely to be a direct target of FGF and Wnt/β-Catenin signalling. (A) Down regulation of *osx* and *pea3* by SU5402 show the same kinetics as monitored by qPCR over a two hour period. (B) Down regulation of *osx* and *TOP*:*GFP* in *hs*:*dkk1* fish shows the same kinetics as monitored by qPCR over a six hour period. (C) A diagram of the *osx* gene centred on the first start codon in exon 1 (white box). Light blue shading shows conservation between zebrafish and medaka genomic sequence (http://genome.ucsc.edu/). Putative Lef/Tcf, Ets1/2 and Runx2 binding sites are indicated above. The amplicons used for ChIP analysis are indicated below, the numbers represent the approximate centre of the amplicon in relation to the start. (D, E) β-Catenin (at 53hpf) and Ets1/2 (at 53hpf) preferentially bind to *osx* genomic sequences when compared to the unrelated gene *her9*. The input bar is the ratio of *her9* amplicon to *osx* amplicon before antibody pull down and the ChIP bar is that ratio after pull down. For normalisation, the input ratio of *her9* to *osx* amplicon is set to 1. Panel E also shows ChIP from fish treated from 51-53hpf with SU5402 which have a mild reduction in Ets1/2 binding activity. p<0.015 for β-CatChIP at osx1146 and p<0.001 for EtsChIP at osx1146.

The activation of *osx* expression by FGF and Wnt/β-Catenin signalling is relatively rapid, suggesting that it may be a direct target of these pathways. To further test this possibility, we performed time courses to compare the kinetics of *osx* regulation to a known target gene. For FGF signalling we treated embryos with SU5402 from 0 to 2 hours and tracked *osx* expression relative to that of *pea3* (Fig 5A). For Wnt/β-Catenin signalling we compared *osx* to the *TOP*:*GFP* transgene after over expression of *dkk1* (Fig 5B). The *TOP*:*GFP* transgene contains a β-Catenin responsive promoter that drives GFP expression [57]. In both situations *osx* down regulation matched that of the known target gene, suggesting that *osx* is a transcriptional target of both pathways.

We next used antibodies to β-Catenin to test whether conserved regions of the *osx* genomic sequence (http://genome.ucsc.edu/) are enriched by ChIP (β-CatChIP). We tested three regions (approximate positions at -13600, -8250 and +97 in relation to the first start codon) and based upon semi-quantitative PCR results (data not shown) focused on the conserved region closest to the start codon. We designed 4 amplicons in this region and performed ChIP with DNA from the *hs*:*dkk1* and *hs*:*wnt8a* lines (Fig 5C and 5D and S4A Fig). We found that at all locations, pull-down by β-Catenin was sensitive to *dkk1* and *wnt8a* over expression (S4B Fig). The most strongly enriched amplicon is at position +1146 and intriguingly this region is close to a putative Runx2 binding site at +824 and is outside of the conserved region of intron 1(Fig 5C).

The mitogen-activated kinase pathway is primarily activated by the FGF receptor in the embryo, and Ets (v-ets erythroblastosis virus E26 oncogene homolog) transcription factors are activated in response to the FGF/Mapk pathway [58–63]. Consistent with a role in *osx* regulation, the Ets factors *pea3*, *erm*, *ets1a* and *ets2* are all expressed in the region of the developing skeleton at 54hpf (S1 and S5 Figs). To test whether Ets factors bind to *osx* intron 1, we took advantage of a cross-reactive Ets1/2 antibody to do ChIP (EtsChIP). We found that as with β-CatChIP, EtsChIP preferentially enriched the +1146 amplicon (Fig 5E). Furthermore enrichment was slightly reduced in the presence of SU5402 suggesting that the ability of Ets1/2 to bind this region is partly FGF dependent. The qPCR time course and ChIP experiments support the model that both FGF and Wnt/β-Catenin directly activate *osx* expression via a shared intronic cis-regulatory module.

### Crosstalk between the Wnt/β-Catenin and FGF pathway

**Fig 6:**
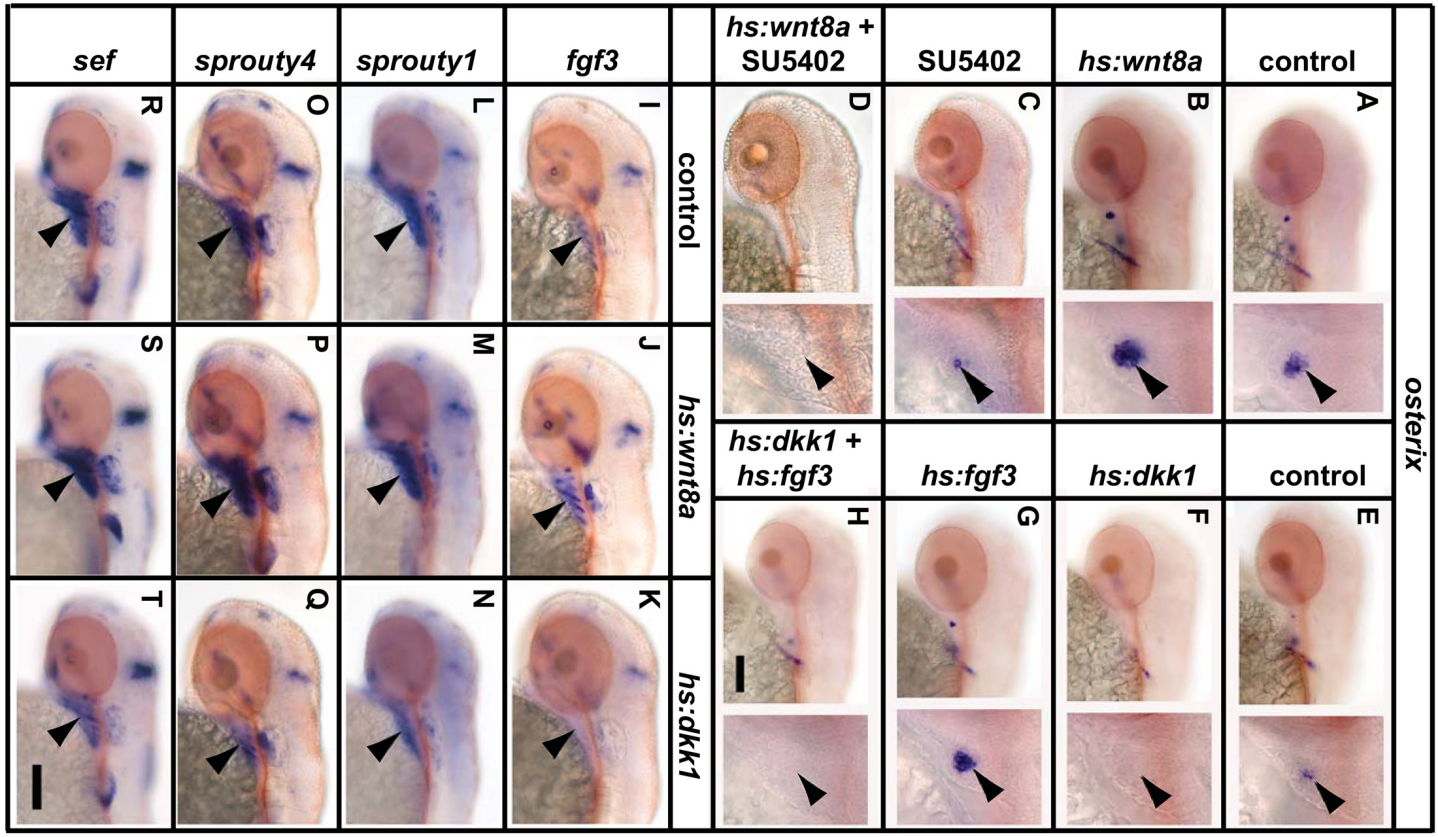
FGF and Wnt/β-Catenin signalling interact to regulate osteoblast differentiation. (A-D) Larvae were heat shocked at 48hpf, treated with SU5402 at 50hpf and fixed at 52hpf. Expression of *osx* is reduced after SU5402 (C) treatment and increased in *hs*:*wnt8* larvae (B). Combined SU5402 and *hs*:*wnt8* treatment still results in reduced expression of *osx* (D). (E-H) Larvae were heat shocked at 49hpf and fixed at 52hpf. Expression of *osx* is reduced in *hs*:*dkk1* larvae (F) and increased in *hs*:*fgf3* larvae (G). Expression is reduced in the combined treatment (H). (I-T) *fgf3*, *sprouty1*, *sprouty4* and *sef* expression is inhibited by *dkk1* over expression (K, N, Q, T) and activated by over expression of *wnt8* (J, M, P, S). Arrowheads point to the opercle in the high magnification images to the right of each panel. Scale bars = 200μM.

Having established that both pathways are likely to act in parallel via intron 1, we wondered whether there is any additional crosstalk between the two pathways which may modify *osx* expression. We first performed epistasis to see whether FGF and Wnt/β-Catenin act sequentially. We combined SU5402 and *hs*:*wnt8a* treatments and found that *wnt8a* activation of *osx* expression is blocked by SU5402 and is therefore FGF dependent (Fig 6A–6D). This would suggest that FGF acts downstream of Wnt/β-Catenin signalling. However, when we combined *hs*:*dkk1*with *hs*:*fgf3* we found that *fgf3* activation of *osx* is dependent upon Wnt/β-Catenin signalling (Fig 6E–6H). Together these data indicates that neither pathway is sufficient to induce *osx* expression on its own. One model to explain these results is that ETS factors and β-Catenin interact in the nucleus to form a complex on the *osx* gene.

We next checked to see whether Wnt/β-Catenin affects activity of the FGF pathway and vice versa. We found that *wnt8a* over expression upregulates components of the FGF pathway while *dkk1* downregulates the same components (Fig 6I–6T). As many components of the FGF pathway are themselves regulated by FGF signalling (through negative feed back), it is difficult to say conclusively that Wnt/β-Catenin is having a positive affect on the pathway as a whole. However, given the timing of the experiments it seems likely that that Wnt/β-Catenin signalling has the capacity to indirectly influence *osx* expression by increasing FGF activity. To test whether a reciprocal interaction takes place, we tested whether expression of components of the Wnt/β-Catenin pathway are affected by SU5402 treatment, *hs*:*fgf3* or *hs*:*dnfgfr1* treatment (S6 Fig). None of these treatments altered expression of *wif*, *axin2*, *lef1* or *tcf7* suggesting that FGF signalling does not modulate the activity of the Wnt/β-Catenin pathway in this context.

## Discussion

**Fig 7:**
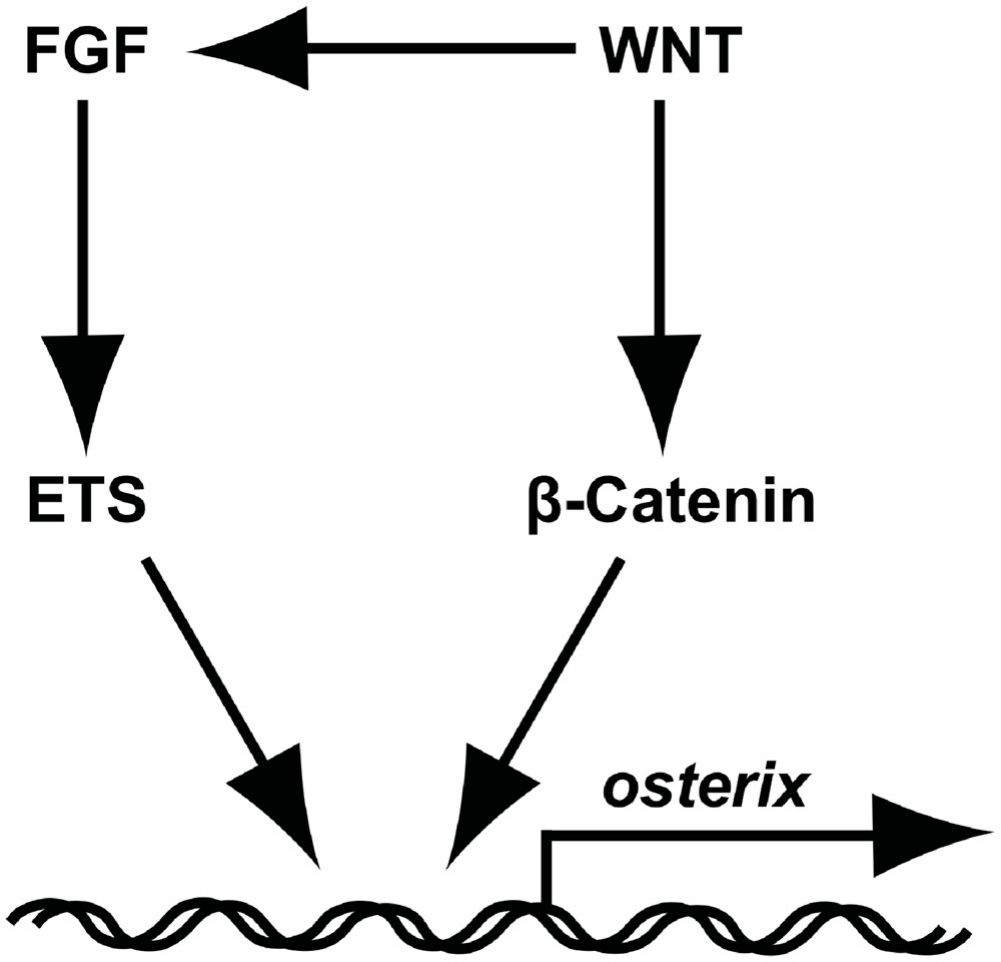
A model for how interactions between FGF and Wnt/β-Catenin signalling pathways interact to regulate *osx* transcription.

Specification of the osteoblast fate choice in mesenchymal stem cells is a multistep process which involves several developmental signalling pathways. Here we investigate this process in the developing facial skeleton and show that both the FGF and Wnt/β-Catenin pathways act on osteoblast precursors to promote bone formation. We have found that manipulation of both pathways has an immediate and strong effect on *osx* expression while having a lesser effect on *runx2* and bone matrix genes. These finding suggest that these pathways both act predominantly at an intermediate level of osteoblast specification after recruitment to the osteoblast lineage. We show that FGF and Wnt/β-Catenin dependant expression of *osx* has the same kinetics as that of known read-outs suggesting that *osx* is a direct target of both pathways. To identify the precise mechanism of action, we have performed ChIP experiments and identified a region of the *osx* first intron that is bound by β-Catenin and ETS1/2 in pull down experiments. Thus we have demonstrated that both pathways are likely to interact at the level of *osx* gene transcription to modulate osteoblast differentiation in vivo (Fig 7). In addition to this interaction on the DNA, we have also found that there is a second potential level of regulation by which the Wnt/β-Catenin pathway acts to modulate activity of the FGF pathway during facial skeletal development.

Many studies on the roles of these pathways in skeletogenesis have not yet revealed a clear picture of how they interact to coordinate osteoblast differentiation. These conflicting results may be explained in part by complex feedback loops and redundancies that may obscure results. Our study aimed to identify the earliest responses to signalling perturbation to help to tease apart these interactions and give a clearer picture of the direct effects of these pathways. We have also utilised an in vivo system to avoid artefacts that are inherent in cell-based studies. One surprising outcome of our study is the relatively weak influence that the Wnt/β-Catenin and FGF pathways exert on *runx2a/b* expression. A consensus of studies in mammals have shown that both pathways activate *runx2* both in vivo and in vitro (reviewed in [64]). This difference may be because our study focuses upon the head skeleton which is primarily neural crest derived, or may indicate a more fundamental difference between zebrafish and mammalian osteoblast differentiation.

One fundamental question in skeletal biology is how signalling pathways regulate the morphogenesis of bones in the early embryo. One recent study showed that *indian hedgehog a* (*ihha*) drives proliferation of cells surrounding the distal edge of the opercle and in *ihha* mutants morphogenesis of the opercle is disrupted[65]. Consistent with this role for Hedgehog signalling, another study has shown that ectopic bone forms on the opercle in *patched1* mutants in which Hedgehog signalling is elevated[66]. Intriguingly, neither study found that Hedgehog signalling regulates expression of *runx2a*, *runx2b* or *osx* during opercle osteoblastogenesis suggesting that *ihha* may act downstream of the Wnt/β-Catenin and FGF pathways. In the future, it will be interesting to identify which FGF and Wnt ligands are expressed around the opercle during this time and to determine how their activity is coordinated with that of *ihha* to the shape the opercle.

## Supporting Information

S1 Fig Inhibition and over activation of FGF signalling have optimal effects 3 hours after the treatment. (A-H) Inhibition of FGF signalling, using SU5402 treatment or *hs*:*dnfgfr1* larvae resulted in strong down regulation of *pea*3 and *erm* expression 3 hours after the treatment (B, C, F, G). Over activation of FGF signalling in *hs*:*fgf3* larvae resulted in strong upregulation of *pea3* and *erm* after 3 hours (D, H). (I-P) 6 hours after the treatment, inhibition of FGF signalling still resulted in slight down regulation of *pea3* and *erm* expression (J, K, N, O) whereas expression was unchanged in *hs*:*fgf3* larvae (L, P). Scale bar = 200μM.
(PDF)

S2 Fig Neither FGF nor Wnt signalling affect *col1a2* expression. (A-C) Larvae were treated at 60hpf and fixed 3 hours later. Expression of *col1a2* is unaffected 3 hours after inhibition (SU5402) or over activation (*hs*:*fgf3*) of FGF signalling. (D-F) Larvae were treated at 60hpf and fixed 12 hours later. Inhibition (*hs*:*dkk1*) or over activation (*hs*:*wnt8*) of Wnt/β-Catenin signalling also does not affect expression of *col1a2*. Arrowheads point to the opercle in the high magnification images to the right of each panel. Abbreviations: cl = cleithrum. Scale bar in F = 200μM.
(PDF)

S3 Fig Wnt/β-Catenin signalling regulates *wif1* and *axin2* expression. **(A-P)** Larvae were treated from 48hpf to 56hpf. Expression of the known Wnt/β-Catenin targets *wif1* and *axin2* is strongly reduced 8 hours after inhibition (*hs*:*dkk1*; D, H, L, P) or increased after over activation (*hs*:*wnt8*; B, F, J, N) of Wnt/β-Catenin signalling.
(PDF)

S4 Fig β-CatChIP preferentially enriches for the +1146 amplicon and is sensitive to Wnt/β-Catenin signalling. β-CatChIP was performed in *hs*:*dkk1* and *hs*:*wnt8* lines heat shocked at 48hpf then fixed at 54hpf and 60hpf respectively. The ratio of the control amplicon *her9* to the *osx* amplicon in the input was used to normalise the levels after ChIP. (A) Shows that -962, +896 and +1146 all show enrichment after ChIP with +1146 being the highest. (B) β-CatChIP is sensitive to Wnt/β-Catenin signalling. Transgenic ChIP is compared directly to sibling ChIP to show that there is on average a 20% reduction in pull down efficiency in *hs*:*dkk1* fish and a 1.5 fold increase in efficiency in *hs*:*wnt8* fish.
(PDF)

S5 Fig Expression of the Ets factors *elk1*, *ets1a* and *ets2*. (A-C) All three factors are expressed in regions around the developing bone at 54hpf, with *ets1a* and *ets2* showing the highest expression.
(PDF)

S6 Fig Expression of Wnt/β-Catenin target genes is unaffected by FGF signalling. (A-P) Larvae were heat shocked or treated with SU5402 at 48hpf and fixed at 51hpf. Expression of *wif*, *axin2*, *lef1* and *tcf7* is unaffected by inhibition (*hs*:*dnfgfr1*) or over activation (*hs*:*fgf3*) of FGF signalling. Scale bar = 200μM.
(PDF)

S1 Table Sequences of primers used in this study. (DOCX)
