# Supplementary figures and images for: Expression of osterix Is Regulated by FGF and Wnt/β-Catenin Signalling during Osteoblast Differentiation
Source: PLoS One. 2015 Dec 21;10(12):e0144982. doi: 10.1371/journal.pone.0144982 (PMC4686927; doi:10.1371/journal.pone.0144982)

Figure S1

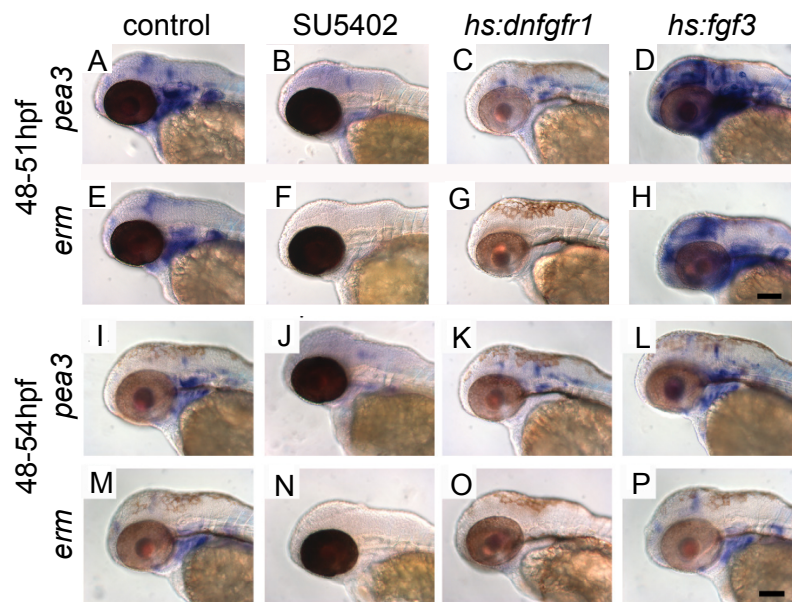

Supplement: S1 Fig — (A-H) Inhibition of FGF signalling, using SU5402 treatment or hs:dnfgfr1 larvae resulted in strong down regulation of pea3 and erm expression 3 hours after the treatment (B, C, F, G). Over activation of FGF signalling in hs:fgf3 larvae resulted in strong upregulation of pea3 and erm after 3 hours (D, H). (I-P) 6 hours after the treatment, inhibition of FGF signalling still resulted in slight down regulation of pea3 and erm expression (J, K, N, O) whereas expression was unchanged in hs:fgf3 larvae (L, P). Scale bar = 200μM. (PDF) [file pone.0144982.s001.pdf]

## Figure S2

Felber et al, Submitted  
FGF and WNT regulate osteoblast differentiation

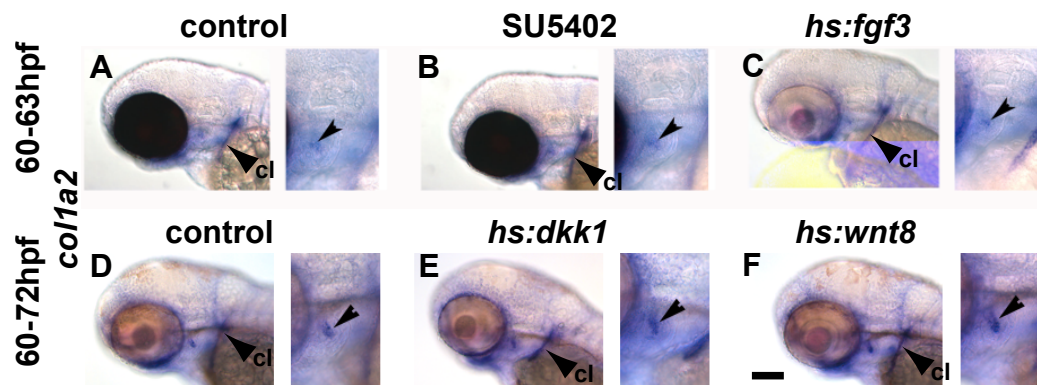

Supplement: S2 Fig — (A-C) Larvae were treated at 60hpf and fixed 3 hours later. Expression of col1a2 is unaffected 3 hours after inhibition (SU5402) or over activation (hs:fgf3) of FGF signalling. (D-F) Larvae were treated at 60hpf and fixed 12 hours later. Inhibition (hs:dkk1) or over activation (hs:wnt8) of Wnt/β-Catenin signalling also does not affect expression of col1a2. Arrowheads point to the opercle in the high magnification images to the right of each panel. Abbreviations: cl = cleithrum. Scale bar in F = 200μM. (PDF) [file pone.0144982.s002.pdf]

**Figure S3**

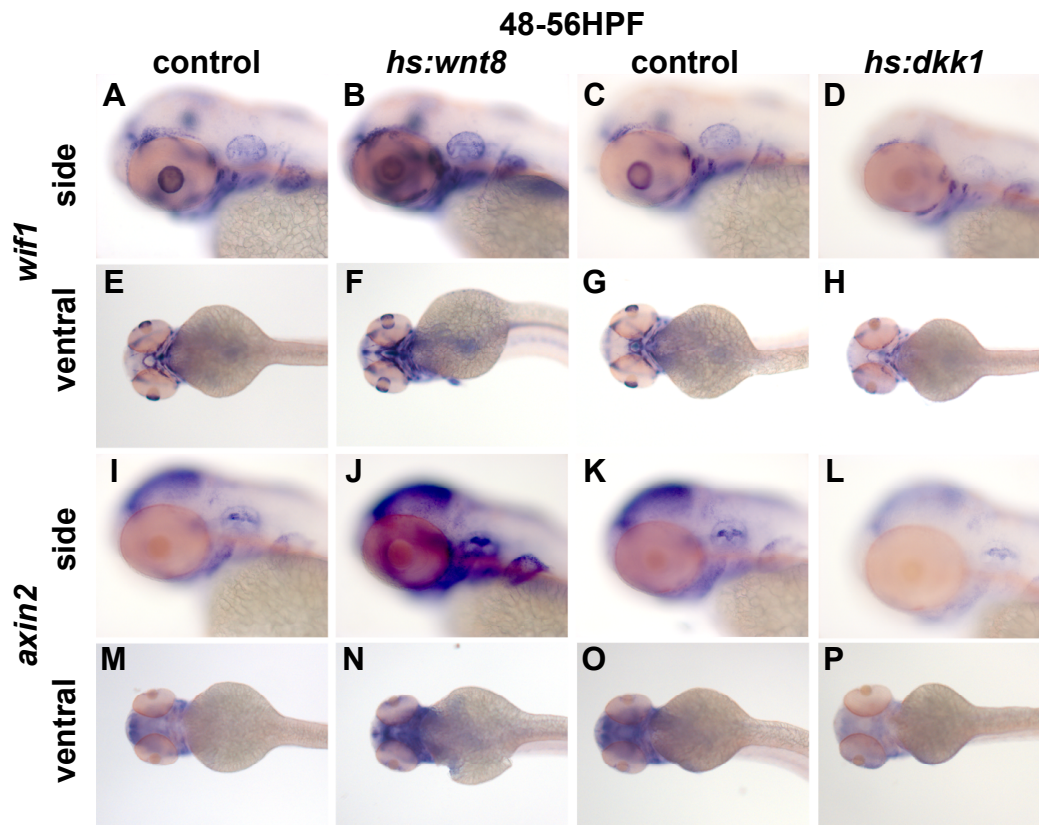

Supplement: S3 Fig — (A-P) Larvae were treated from 48hpf to 56hpf. Expression of the known Wnt/β-Catenin targets wif1 and axin2 is strongly reduced 8 hours after inhibition (hs:dkk1; D, H, L, P) or increased after over activation (hs:wnt8; B, F, J, N) of Wnt/β-Catenin signalling. (PDF) [file pone.0144982.s003.pdf]

Figure S4

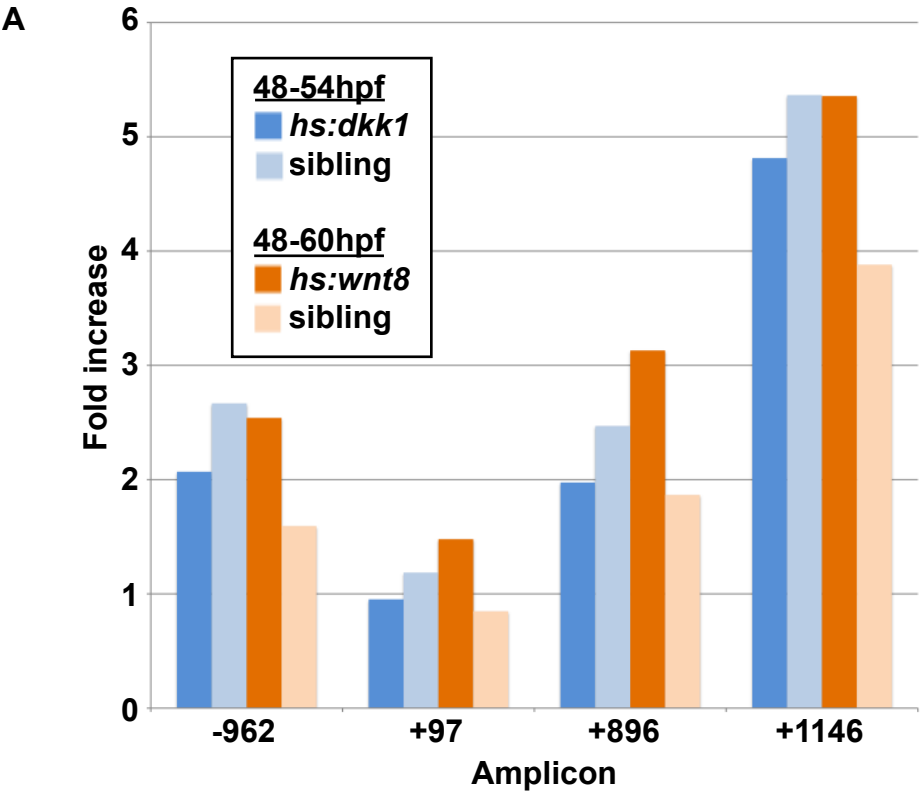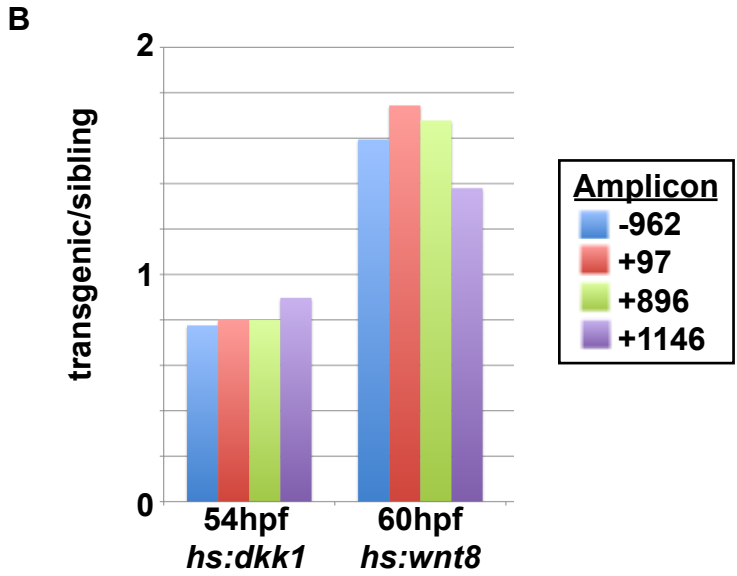

Supplement: S4 Fig — β-CatChIP was performed in hs:dkk1 and hs:wnt8 lines heat shocked at 48hpf then fixed at 54hpf and 60hpf respectively. The ratio of the control amplicon her9 to the osx amplicon in the input was used to normalise the levels after ChIP. (A) Shows that -962, +896 and +1146 all show enrichment after ChIP with +1146 being the highest. (B) β-CatChIP is sensitive to Wnt/β-Catenin signalling. Transgenic ChIP is compared directly to sibling ChIP to show that there is on average a 20% reduction in pull down efficiency in hs:dkk1 fish and a 1.5 fold increase in efficiency in hs:wnt8 fish. (PDF) [file pone.0144982.s004.pdf]

## Figure S5

Felber et al, Submitted  
FGF and WNT regulate osteoblast differentiation

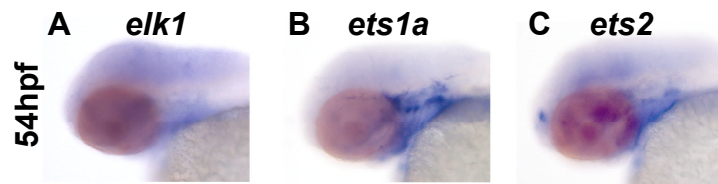

Supplement: S5 Fig — (A-C) All three factors are expressed in regions around the developing bone at 54hpf, with ets1a and ets2 showing the highest expression. (PDF) [file pone.0144982.s005.pdf]

Figure S6

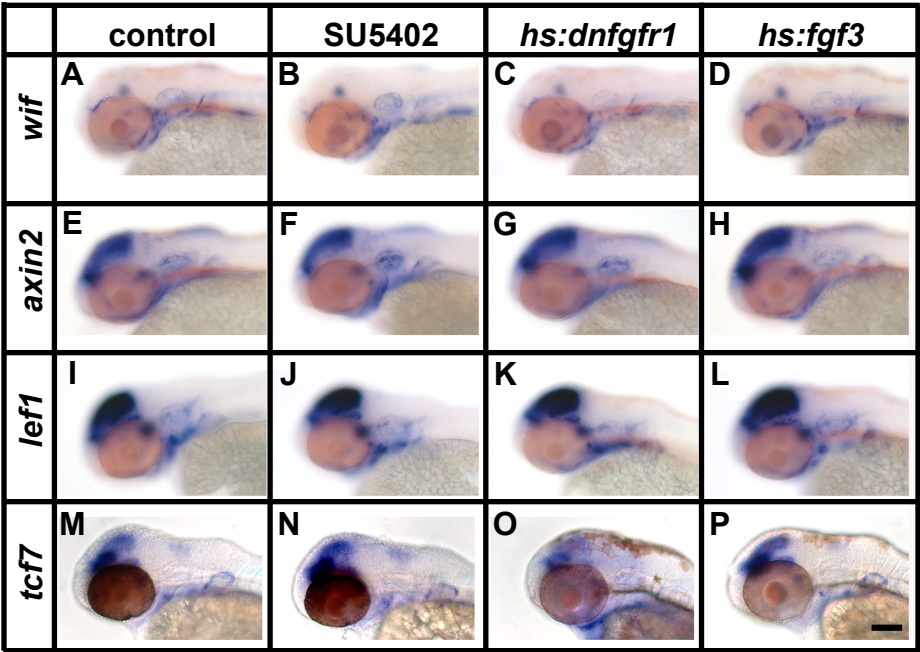

Supplement: S6 Fig — (A-P) Larvae were heat shocked or treated with SU5402 at 48hpf and fixed at 51hpf. Expression of wif, axin2, lef1 and tcf7 is unaffected by inhibition (hs:dnfgfr1) or over activation (hs:fgf3) of FGF signalling. Scale bar = 200μM. (PDF) [file pone.0144982.s006.pdf]
