# Supplementary material for: Expression of osterix Is Regulated by FGF and Wnt/β-Catenin Signalling during Osteoblast Differentiation
Source: PLoS One. 2015 Dec 21;10(12):e0144982. doi: 10.1371/journal.pone.0144982 (PMC4686927; doi:10.1371/journal.pone.0144982)
Supplement: S1 Table — (DOCX) [file pone.0144982.s007.docx]

**S1 Table. Sequences of primers used in this study.**

**Primer sequences (SYBR Green)**

| **Gene** | **Forward Primer** | **Reverse Primer** |
| --- | --- | --- |
| *osx* | ctggatacgccgctgggtcta | tgtggacaggtttcttccgca |
| *pea3* | tgagtcgctctttgcgttact | ttatcaaagcttctggctcac |
| *pgk-1* | caaagttgtagaagcgaccaaa | gctgaccttgtcctcagtgtc |

**Primer sequences (TaqMan)**

| **Gene** | **Forward Primer** | **Labelled Probe** | **Reverse Primer** |
| --- | --- | --- | --- |
| *osx* | gccgctgggtctacca | ccgggaggtcttcttg | cctgacaattcgggcaatcg |
| *pgk-1* | gacaaagttgtagaagcgaccaaaa | tcccccaccgataatg | cttggcacagcaggtagct |
| *top-gfp* | ggtggaggagcaggatgatg | cacgctgcccatgtct | gcagggtgacggtccat |

**ChIP Primers**

| **Position** | **Forward Primer** | **Reverse Primer** |
| --- | --- | --- |
| *+ 1146* | cacatttaagctccacgcg | tgtgatgatatttcccgctg |
| *+ 896* | agggacagttcaccccaaat | tgtttcaaacctccatttcttg |
| *+ 97* | gcgatctcacagctgctctt | tcaaacatgaacatacttcacttca |
| *- 962* | gacgcttgtgagtcctgctag | cacgtgacgaatatcccaacca |
| *her9* | tattggctgaagcgagtgtg | gggacgatcacgagagagag |
